# ASO Author Reflections: Isolated Margin Positivity in Localized Pancreatic Ductal Adenocarcinoma and Operative Margin Revision

**DOI:** 10.1245/s10434-024-15658-2

**Published:** 2024-06-21

**Authors:** Mohamedraed Elshami, Lee M. Ocuin

**Affiliations:** Division of Surgical Oncology, Department of Surgery, University Hospitals Cleveland Medical Center, Cleveland, OH USA

## Past

Retrospective analyses investigating intraoperative margin revision in patients with pancreatic ductal adenocarcinoma (PDAC) undergoing pancreatectomy have yielded conflicting results. Some studies have reported that additional resection to achieve an R0 margin on permanent section was not associated with higher overall survival (OS) or recurrence-free survival (RFS).^1,2^ Other studies have suggested that intraoperative revision of positive neck margins may lead to prolonged survival.^3^ To date, no studies have examined the frequency of isolated margin positivity (IMP) in patients with localized PDAC who underwent pancreatectomy (pancreatoduodenectomy, distal pancreatectomy, or total pancreatectomy) and determined the fraction of patients that would potentially derive a survival benefit from revising positive neck or surgical margins; conceptually, these would be patients with no adverse pathologic features (nodal positivity, perineural invasion, and lymphovascular invasion) besides margin positivity, and revision of this isolated margin would result in absence of any adverse pathologic feature and theoretically higher survival.

## Present

Malleo and colleagues recently reported on 671 patients with PDAC who received neoadjuvant therapy and underwent pancreatoduodenectomy. Intraoperative frozen-section analysis was performed, and margin revision of an initially positive neck margin to negative was not associated with improvements in overall or recurrence-free survival.^4^

In the current study, we demonstrated the rarity of IMP, which occurs in < 1% of patients in our institutional cohort and was similarly rare in patients within the National Cancer Database (NCDB) cohort.^5^ Within our institutional cohort, we estimated the fraction of patients potentially benefiting from revision of an isolated positive margin (any) or neck margin was approximately 1 in 18,500 and 1 in 100,000 patients, respectively. Similarly, 1 in 25,000 patients in the NCDB cohort may derive a survival benefit from revision of a positive margin (the NCDB does not report margin location). Isolated margin positivity was associated with lower OS compared with no adverse pathological features but was associated with higher OS compared with margin positivity and additional adverse pathologic features.

## Future

As is usually the case, a well-designed randomized controlled trial is the only way to test rigorously the oncologic benefits and potential risks associated with revising positive margins during pancreatectomy. However, based on our report and the rarity of IMP, the feasibility of such a study is questionable. There is a need for the development of novel intraoperative imaging techniques or molecular assays to identify accurately positive neck margins in real-time, which could facilitate more precise intraoperative decision making and improve patient outcomes. As a field, we have likely “maxed out” surgical approaches and technical aspects of management of localized PDAC in light of the constant debates about open versus minimally invasive pancreatectomy and which approach is better—a search that has continued 30 years after the first laparoscopic pancreatoduodenectomy was performed. Identification of novel therapeutic targets and development of novel, more effective drugs through multidisciplinary collaborative efforts will provide the most benefit for the treatment of this biologically aggressive malignancy.

## References

[1] Hernandez J, Mullinax J, Clark W, et al. Survival after pancreaticoduodenectomy is not improved by extending resections to achieve negative margins. *Ann Surg*. 2009;250(1):76–80. 10.1097/SLA.0b013e3181ad655e. 19561479 10.1097/SLA.0b013e3181ad655e

[2] Kooby DA, Lad NL, Squires MH 3rd, et al. Value of intraoperative neck margin analysis during Whipple for pancreatic adenocarcinoma: a multicenter analysis of 1399 patients. *Ann Surg*. 2014;260(3):494–501. 10.1097/SLA.0000000000000890. 25115425 10.1097/SLA.0000000000000890

[3] Zhang B, Lee GC, Qadan M, et al. Revision of pancreatic neck margins based on intraoperative frozen section analysis is associated with improved survival in patients undergoing pancreatectomy for ductal adenocarcinoma. *Ann Surg*. 2021;274(2):e134–42. 10.1097/SLA.0000000000003503. 31851002 10.1097/SLA.0000000000003503

[4] Malleo G, Lionetto G, Crippa S, et al. Reappraising the role of intraoperative neck margin revision in post-neoadjuvant pancreatoduodenectomy for pancreatic ductal adenocarcinoma: a multi-institutional analysis. *Ann Surg*. 2024. 10.1097/SLA.0000000000006322. 10.1097/SLA.0000000000006322 38708617

[5] Elshami M, Wu VS, Stitzel HJ, et al. To revise or not to revise? Isolated margin positivity in localized pancreatic ductal adenocarcinoma. *Ann Surg Oncol*. 2024. 10.1245/s10434-024-15616-y. 10.1245/s10434-024-15616-y PMC11300499 38896228

